# Synthetic Blocks for Bone Regeneration: A Systematic Review and Meta-Analysis

**DOI:** 10.3390/ijms20174221

**Published:** 2019-08-28

**Authors:** Margherita Tumedei, Paolo Savadori, Massimo Del Fabbro

**Affiliations:** 1 University “Gabriele D’Annunzio” of Chieti-Pescara, 66100 Chieti, Italy; 2 IRCCS Istituto Ortopedico Galeazzi, 20161 Milan, Italy; 3 Department of Biomedical, Surgical, and Dental Sciences, Università degli Studi di Milano, 20161 Milan, Italy

**Keywords:** animal models, biomaterials, block graft, bone graft, bone regeneration, bone substitutes, histological analysis, synthetic biomaterials, systematic review

## Abstract

This systematic review is aimed at evaluating the effectiveness of synthetic block materials for bone augmentation in preclinical in vivo studies. An electronic search was performed on Pubmed, Scopus, EMBASE. Articles selected underwent risk-of-bias assessment. The outcomes were: new bone formation and residual graft with histomorphometry, radiographic bone density, soft tissue parameters, complications. Meta-analysis was performed to compare new bone formation in test (synthetic blocks) vs. control group (autogenous blocks or spontaneous healing). The search yielded 214 articles. After screening, 39 studies were included, all performed on animal models: rabbits (*n* = 18 studies), dogs (*n* = 4), rats (*n* = 7), minipigs (*n* = 4), goats (*n* = 4), and sheep (*n* = 2). The meta-analysis on rabbit studies showed significantly higher new bone formation for synthetic blocks with respect to autogenous blocks both at four-week (mean difference (MD): 5.91%, 95% confidence intervals (CI): 1.04, 10.79%, *p* = 0.02) and at eight-week healing (MD: 4.44%, 95% CI: 0.71, 8.17%, *p* = 0.02). Other animal models evidenced a trend for better outcomes with synthetic blocks, though only based on qualitative analysis. Synthetic blocks may represent a viable resource in bone regenerative surgery for achieving new bone formation. Differences in the animal models, the design of included studies, and the bone defects treated should be considered when generalizing the results. Clinical studies are needed to confirm the effectiveness of synthetic blocks in bone augmentation procedures.

## 1. Introduction

The restoration of damaged tissues represents the main goal of contemporary medical research [1]. The ideal goal is a newly developed tissue with the same immunological, functional, structural, and mechanical characteristics as the native one [2,3]. In oral and maxillofacial surgery, achieving bone regeneration is necessary to allow a predictable prosthetic rehabilitation supported by dental implants in the case of severe bone defects [4,5].

Though autologous bone is still considered the best grafting material for bone regeneration purposes, the main clinical drawbacks are the limited quantity available and the morbidity of the donor site, which requires careful management [6]. In order to address such issues, bone regeneration procedures in oral and maxillo-facial surgery take advantage of mechanical and biological properties of various types of bone substitute scaffolds to improve the healing process of damaged hard tissues [7].

Many biomaterials and surgical techniques have been developed and may be safely used for bone regeneration [8,9]. The selection of the appropriate biomaterial cannot neglect the knowledge of its properties and behavior in order to achieve a predictable result and clinical success of the regenerative procedure. The three-dimensional structure of the scaffolds plays a crucial key role in bone regeneration [3,10]. In this way, biomaterials should provide a provisional tridimensional guide and a mechanical support to the cells active in the early stages of bone regeneration [10,11]. Porosity, topography, chemical surface composition, 3D architecture, immunogenicity, and mechanical features significantly influence the bone formation [12].

The integration of a biomaterial with the host tissue occurs through different mechanisms, such as osteogenesis, osteoconduction, and osteoinduction [13]. Even though autologous bone shows all these characteristics and is considered as the golden standard of grafting material, several drawbacks have limited its use in relation to the morbidity of the donor site [14].

Therefore, different osteoconductive biomaterials have been developed and several studies showed that they may favor cell attachment and proliferation, improving both quantitatively and qualitatively the bone regeneration by promoting physiological healing at the bone defect and forming a mature tissue with adequate mechanical properties [5,9,10]. Engineering of synthetic bone graft substitute has been indicated as a promising alternative to the autograft methods [6,8]. Indeed, synthetic bone graft substitutes have been successfully used for vertical ridge augmentation prior to implant placement.

An ideal synthetic biomaterial should be biocompatible, resorbable, showing a minimal fibrotic reaction, able to undergo remodeling and support new bone formation, cost-effective, and easy to use [15]. From a mechanical point of view, synthetic bone graft substitutes should have a similar strength to that of the cortical/cancellous bone being replaced [16]. Different synthetic biomaterials are currently used in bone regeneration and can be classified into three groups according to their composition: metallic (NiTi and MgF2 alloy), ceramic (Hydroxyapatite (HA, Bioglass), and polymeric (polylactic-co-glycolic acid (PLGA), polycaprolactone (PCL), polyglycolic acid (PGA), polylactic acid (PLA)) [16,17].

The synthetic ceramic biomaterials have good compressive strengths but they are weak in tension and shear, brittle, and fracture-prone on shock loading [18]. On the contrary, synthetic HA in solid block form is difficult to shape, does not permit fibro-osseous ingrowth, and has a much higher modulus of elasticity than bone [19]. Graft microporosity is able to bioactively influence the differentiation and functional activity of cells in vitro involved in the physiological events related to the osteogenic processes [20,21]. Polymeric materials, such as acrylic bone cements, have been proposed for the treatment of bone defects in orthopedics and implantology, due to their favorable microstructure and bioactivity properties [22].

Moreover, the metallic materials application should be limited to a contained area due to the modulus of elasticity higher than the bone [19]. An ideal biomaterial should have similar modulus of elasticity to that of bone in an attempt to prevent stress shielding as well as maintain adequate toughness to prevent fatigue fracture under cyclic loading [7,8,9].

The polymeric materials are more beneficial than others because of their biocompatibility, mechanical properties, microstructure, and degradation rate, and these properties can even be precisely controlled by composition and fabrication of scaffold polymer materials [23].

The aim of this study was to perform a systematic literature review of preclinical in vivo studies with the objective to determine which are the indications deriving from research of the most effective synthetic bone block materials for bone augmentation techniques. The PICO (Problem, Intervention, Comparison, Outcome) question of this review was: Are synthetic bone blocks effective for the treatment of bone defects with respect to other graft materials or ungrafted sites?

## 2. Results

**Figure 1:**
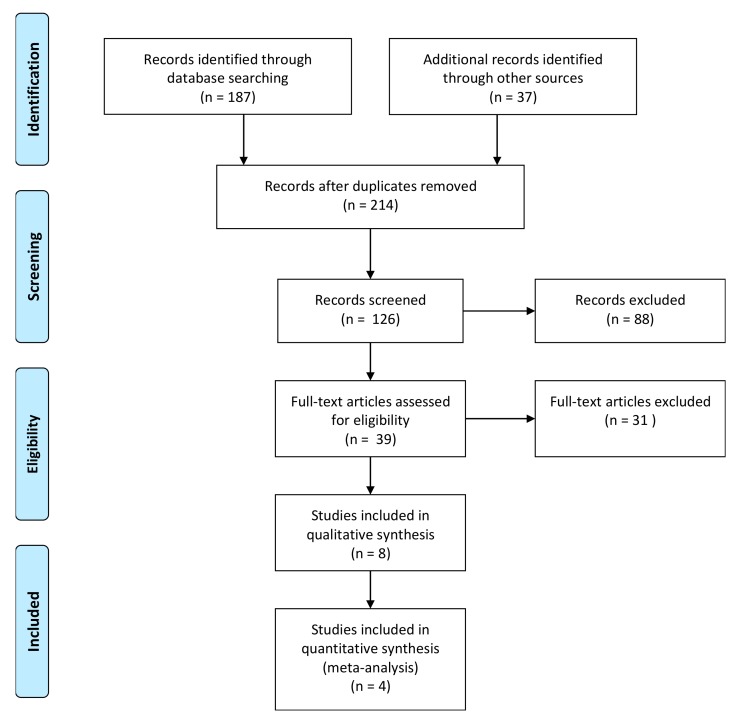
Flowchart of the study selection process (adapted from PRISMA guidelines [24]).

The electronic search procedure is presented in Figure 1. The database analyses generated a total of 182 references. A manual search was also performed and a total of 32 articles were added to the output article list, generating a total of 214 articles retrieved. After title and abstract evaluation, 175 articles were excluded: 2 articles written in Chinese language, 6 clinical case series/case report studies, 49 in vitro articles, 19 literature reviews, 84 articles nonpertinent to the topic, and 15 studies where a combination with autogenous bone particles was used.

At the end of the procedure, 39 articles satisfied the inclusion criteria and were included in this review analysis: 18 studies were performed in a rabbit study model, 4 on dogs, 7 on rats, 4 on minipigs, 4 on goats, and 2 articles on sheep (Figure 1). 

No study on humans was included. The main characteristics of the defect treated, bone graft material characteristics, study outcome, and follow-up time for each animal model are summarized in Table 1, Table 2, Table 3, Table 4, Table 5 and Table 6. Thirty-seven studies (97.3%) performed histologic evaluation of the samples retrieved [18,25,26,27,28,29,30,31,32,33,34,35,36,37,38,39,40,41,42,43,44,45,46,47,48,49,50,51,52,53,54,55,56,57,58,59,60], 26 studies (68.4%) reported histomorphometric analysis, 1 study performed fluorescence microscopy analyses of bone samples [18]. Two studies [47,56] described a scanning electron microscope (SEM) evaluation. Eleven articles [28,29,30,31,32,39,40,51,54,59,61] also performed microcomputed tomography (micro-CT), five a radiographic assessment [18,38,44,55,62], and one article [50] reported an angiogram evaluation. One study also reported X-ray diffraction evaluation [42]. An analytic description of the evaluation techniques is provided in Table 1, Table 2, Table 3, Table 4, Table 5 and Table 6.

### 2.1. Risk-of-Bias Assessment 

**Figure 2:**
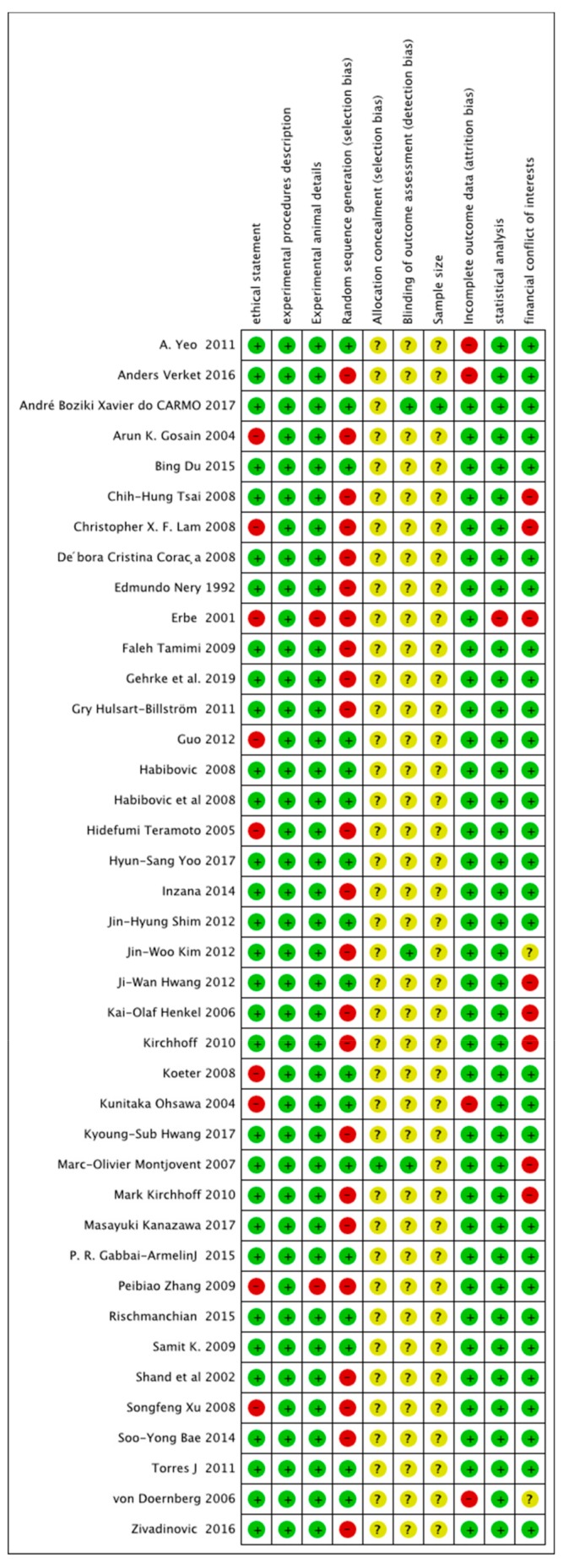
Risk-of-bias graph: review authors’ judgments about each risk-of-bias item presented as percentages across all included studies. + = yes (the criteria was met, green circle); − = no (the criteria was not met, red circle); ? =unclear if the criteria was met or not (yellow circle). The figure was created using RevMan Version 5.3 (The Nordic Cochrane Centre, The Cochrane Collaboration, Copenhagen, 2014).

**Figure 3:**
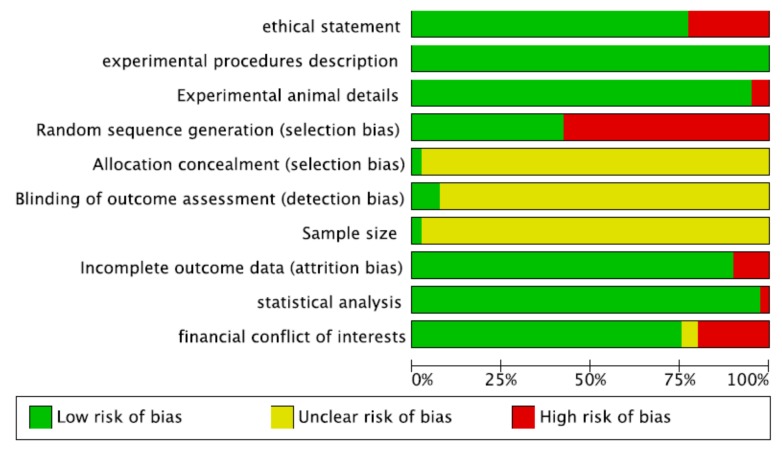
Risk-of-bias summary: review authors’ judgments about each risk-of-bias item for each included study. The colors have the same meaning as in Figure 2. The figure was created using RevMan Version 5.3 (The Nordic Cochrane Centre, The Cochrane Collaboration, Copenhagen, 2014).

Risk-of-bias ratings across all included articles is presented in Figure 2. Only 10 studies [25,30,37,41,55,56,61] were classified as having a low risk of bias, whereas all other included studies presented a high risk of bias, based on the parameters evaluated (Figure 3). 

Among them, the studies selected showed a wide heterogeneity about the study design, analytical methods, and follow-up period. Erbe et al. reported the longest follow-up period in a study on dog bone defects [44]. The unfilled defect represented the most diffused experimental control site of the studies selected for the review.

### 2.2. Meta-Analysis

**Figure 4:**
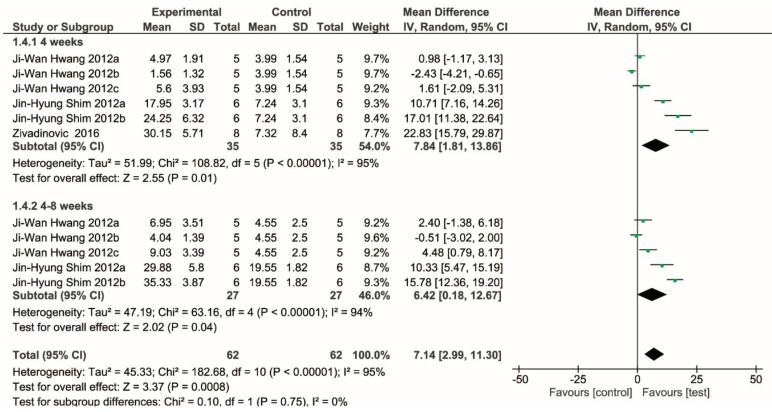
Forest plot showing the meta-analysis of the comparative studies performed in rabbits, with a follow-up of four and eight weeks. The results demonstrate evidence for a significantly higher new bone formation in the group using synthetic blocks. The figure was created using RevMan Version 5.3 (The Nordic Cochrane Centre, The Cochrane Collaboration, Copenhagen, 2014).

Following data extraction, four comparative studies were identified that provided the histomorphometric results of new bone formation in different animal models using synthetic bone blocks versus a control group in which the defect was left to heal spontaneously [26,28,30,41]. In one study, autogenous bone graft was used as the control [27]. The data were grouped according to the follow-up duration: four weeks and longer than four (mostly eight) weeks. Table 7 shows the data extracted from these studies: four studies were performed on rabbits, one each on dog model, rat, sheep, and minipig. Since bone features and healing rate of different animal models present considerable differences, and due to the fact that for some animals only one study was found, a meta-analysis was performed only for studies based on the rabbit model. One of such studies used autogenous bone block as the control [64] and was excluded from the meta-analysis, aiming at comparing synthetic blocks versus unfilled sites. The results of the meta-analysis, synthesized in the Forest plot in Figure 4, showed that there is evidence for a significantly higher new bone formation in the groups using bone blocks as compared to the control group. The difference appears to smoothen slightly at the longer follow-up. It must be noted, however, that a significant heterogeneity was found among studies at both follow-ups. About half of the comparisons showed a nonsignificant effect, while one study reported a higher new bone formation in the control group [45].

### 2.3. Studies Not Included in the Meta-Analysis 

In spite of the large heterogeneity in the choice of the experimental site, from calvaria to long bones, intraoral regions, or other skeletal or ectopic sites, and considering the different animal models, the different materials, and the different techniques to assess the performance of the tested materials, most studies reported favorable results when using synthetic blocks for bone defect regeneration. 

In rabbits, the most used experimental site was the calvaria (12 out of 18 studies), followed by long bone defects (tibia, femur, radius), while one study assessed mandibular defects (Table 1). In one study, the control group, represented by autogenous bone, showed markedly better histomorphometric results than the experimental group [27], while in the other studies, in which the control was the unfilled defect, generally better outcomes were achieved using synthetic bone blocks (Table 7).

In dogs, three studies evaluated intraoral bone defects and one had a cylindrical metaphyseal defect as the model (Table 2). In the study by Rismanchian et al., significantly better results (higher amount of lamellar bone and woven bone) were obtained using blocks of forstrite than using bioglass blocks, and both were better than unfilled controls in maxillary bone defects [41]. In a study on periodontal osseous defects, blocks of Hydroxyapatite/β-tricalciumphosphate (HA/β-TCP) with different macroporosity (ratio from 100/0 to 0/100) and biphasic calcium phosphate ceramic were compared to unfilled controls [43]. After six months, the better results (gain in attachment level and bone defect regeneration) were obtained using the combination 85% HA/15% X.

Seven studies on rats were included (Table 3), of which three were on long bones [45,46,47], two on the calvaria model [48,62], one on a postextraction maxillary socket [50], and one on ectopic bone formation [49]. Some of the studies on long bones and calvaria used PLA (poly(l-lactic acid))-based blocks that were demonstrated to be osteocompatible and to have good osteoconductive properties [46,48,62]. The study of Billström et al. [49] showed that a material containing nanostructured hydroxyapatite and BMP-2 can induce the formation of ectopic bone with a density higher than other materials tested (β-TCP and different forms of HA).

All studies on minipigs were performed on intraoral models (Table 4). The studies by Yeo et al. [51] and Kirchoff et al. [52] investigated mandibular ridge augmentation using autogenous bone block plus collagen membrane as the control. The histomorphometric results of both studies showed clear superiority of the control group at 5/10 weeks [53] and at 24 weeks. The study by Henkel et al. evaluated different graft materials based on CaP fabricated with a sol/gel process at 700 °C versus HA/β-TCP ceramics, or a gelatin sponge in mandibular critical size defects (>5 cm^3^) [52]. The best results in terms of bone formation and material resorption rate were achieved using CaP biomaterials (> 93% bone formation versus < 58% with the classical ceramics), which also showed lower percentage of residual material than HA/β-TCP ceramics after eight months [53].

The four studies performed on the goat model all investigated orthopedic defect types (Table 5). The study of Nandi et al. on radius defects treated with porous bioactive glass blocks, as compared to unfilled defects, showed well-organized and vascularized bone tissue after three months in the test group [55]. Two studies on lumbar transverse process defects showed good osteoinductivity of porous biphasic calcium phosphate ceramics (HA/β-TCP) with up to 12 weeks of orthotopic bone healing [57], and a similar performance of brushite (dicalcium phosphate dehydrate) as compared to monetite (dicalcium phosphate anhydrous) [56] in terms of orthotopic and ectopic bone formation for up to 9 weeks.

Finally, two studies on the sheep model were included (Table 6). One evaluated β-TCP cylinders with different pore sizes (150 nm, 260 nm, 510 nm, and 1220 nm) in methaphysial or epiphysial long bone defects [59]. The best outcomes in terms of new bone formation were obtained with blocks having 260 nm pores. The second study was on the reconstruction of critical-size defects in the calvaria (five defects of 16.8 mm diameter per animal), filled with blocks of four different materials (see Table 6) [60]. After one year of healing, the best result in terms of mean bone replacement, as determined by computed tomography, was achieved in the group using a mixture of 20% hydroxyapatite-cement and 80% TCP (28.5 ± 4.5%).

## 3. Discussion

The purpose of the present investigation was to perform a systematic review and a meta-analysis of in vivo studies that evaluated the efficacy of synthetic scaffolds in block form for bone regeneration. We aimed at gathering the current evidence, assessing biomaterials’ performance and side effects, and detecting knowledge gaps for the design of future clinical trials.

The outcome of the review highlights a wide heterogeneity of the studies included, presenting differences in methodological model, procedural technique, and the type of biomaterials analyzed. The studies selected for the present review included six animal species (rabbit, rat, dog, minipig, goat, and sheep). Each species presents peculiar features, making interspecies comparison, as well as comparison with bone regeneration in humans, quite difficult. 

Using small-size animals like mice, rats, and rabbits carries some advantages, including easy handling, relatively short time for bone healing, and low cost. Although rats are used extensively as models for bone healing, they present consistent differences with human bone anatomy and physiology, making them suitable only as a first-step approach, to demonstrate the biotolerance and safety of the material. [65,66].

Conversely, rabbits have more similarities with human bone anatomy [65]. In particular, the bone macro- and microarchitecture of the rabbit femoral condyle resemble the ones of implantation sites in the human jawbone. For this reason, the rabbit is considered a valid model for preclinical studies on dental implants [67].

As opposed to small animals, large-size animal models have more features in common with human bone anatomy and/or physiology according to the literature [65,66,68]. Actually, small ruminants like goat and sheep are suitable models for investigating orthopedic implants and prostheses due to long bone and joint dimensions similar to humans. Furthermore, load bearing is very similar [65,68]. The best animal model for studying bone regeneration is considered the minipig [69]. They have bone architecture, anatomy, and bone healing rate very close to those of humans [65,70]. Also in dogs, bone composition and structure have many features in common with humans. In the past 30 years, canine models have been often used in periodontology and implant dentistry research [65]. However, using the dog as an experimental model may raise some ethical issues. 

Large-size animal models present further drawbacks as compared to small-size animals, such as high cost, seasonal breeding cycles (sheep), long time for bone healing, and requirement for appropriate instrumentation and facilities [65,69]. 

In conclusion, the differences between the animal species, the size of the defects, and the healing patterns represent sensible variables for the study outcome evaluation. When considering the transferability of the results of the present review on synthetic bone blocks, some further consideration regarding the limitations is needed. In addition to the above comments regarding transferability of some animal models to humans, one should consider that the majority of the studies were performed on experimentally created bone defects in healthy animals. This might produce a different healing response as compared to diseased tissue in which the bone defect may be the consequence of a given disease. In fact, the etiopathogenesis of diseases leading to bone defects creates a tissue environment that is quite different from the one present in an experimentally induced bone defect.

Nevertheless, interesting indications could be drawn from the meta-analysis of studies on the rabbit model, which clearly showed a favorable effect of synthetic blocks, in terms of new bone formation.

Our results confirmed that the biomechanical features of the synthetic bone substitutes could play a key role in bone regeneration, as they showed improved regenerative properties in qualitative and quantitative terms, as compared to sham control groups. In fact, these features suggest that the concept of three-dimensional architecture of the microstructure and the chemical nature of the scaffold could produce a determinant positive influence in the regenerative process and in newly formed bone tissue features. In this way, some features that an ideal biomaterial should possess can be identified [71,72,73]. For example, there should be an adequate porosity ensuring interconnection among pores of an adequate size, in order to allow for diffusion of bone cells and nutrients and exchange of waste products throughout the whole graft. The size of pores should be of at least 100 microns, though >300 microns is the recommended size for allowing neoangiogenesis and formation of new bone [74]. Another important feature is the interface between the material and the environment in which the material is embedded, in terms of both surface area (which is depending on the material porosity) and surface texture. Both are critical for allowing optimal vascular ingrowth, bone cell adhesion, migration, and proliferation. A third important property of the ideal graft material is an adequate mechanical compressive strength and flexibility, in order to absorb loading forces from the surrounding hard and soft tissues, especially in noncontained defects. This is particularly relevant in appositional grafts, like most of the block grafts considered in the present review. Finally, the stability of the biodegradable synthetic biomaterials while promoting bone ingrowth and the substitution of the graft with new bone tissue is another essential feature [6,7,8,9,10,12]. In fact, the biomaterial degradation should not occur faster than replacement with new bone, so that the supporting function is ensured throughout the graft maturation process. For example, Shim et al. showed in rabbit a significant increase of the new bone density in polycaprolactone/polylactic-co-glycolic acid scaffold blended with tri-calcium phosphate (a material with considerable elastic properties) at four and eight weeks compared to the unfilled sites [30].

The surface chemistry of the bone block could also play an important role in relation to the biomechanics and substitution capability of the biomaterial with the new bone, the physio-biology of the regenerated tissues, and the healing time of the graft [64].

On the contrary, Gabbai-Armelin et al. [45] reported in rats an earlier and more consistent new bone formation in the unfilled tibial defects at 15 days in comparison to the sites grafted with bioglass, but no statistical differences in the subsequent experimental follow-up times of the study.

Rismanchian et al. [41] tested different synthetic scaffolds in dogs and highlighted that a nanocomposite forstrite graft (Mg_2_SiO_4_) showed an increased capability of volume maintenance and lamellar bone formation after the healing time if compared with the unfilled site in postextractive socket defect, with a high bonding strength and fracture toughness. Previous studies confirmed that the regenerative outcome of synthetic blocks depends on a combination of physico-chemical properties that provide cell adhesion and proliferation, tissue ingrowth, and substitution with new vital bone [75].

Tamimi et al. [27] reported no statistically significant differences in vertical bone augmentation with autologous bone grafts and monetite blocks at eight weeks of healing in the rabbit calvarial model.

All of these studies’ findings suggest that the scaffold should be chosen in relation to its nature, features, and biology, according to specific surgical applications and indications, in order to optimize and improve the clinical outcome.

As confirmed by recent literature, the optimal properties of the synthetic block depend on the matching of biomechanical features of the scaffold, the regenerated bone, and the native tissue [76].

About the structure of biomaterials, several authors proposed that application of the graft in form of either bone block or particulate may be equally effective for the treatment of bone atrophy [33,77,78,79,80].

Moreover, the surface chemistry of the bone block may play an important role in relation to the new bone formation, improving the amount and composition of host protein adhering to a surface. In this way, physical or chemical modifications have been proposed to improve biomaterial compatibility [81].

Troeltzsch et al. [82] reported that vertical augmentation was substantially higher when space-making barrier materials such as titanium meshes were used. However, this was also associated with a strong increase in complication rate [71]. When using block grafts, a higher horizontal gain, by approximately 1 mm, was achieved [71].

Tolstunov et al. [83] classified the horizontal bone atrophies in the horizontal bone defect of Class III–V (between 2 and 6 mm of thickness), suggesting that satisfying results may be achieved with the use of the bone block graft techniques.

The treatment of the vertical and combined (vertical plus horizontal) defects with bone blocks may present an increased difficulty also regarding the soft-tissue management. Clinically, it was reported to be less predictable than when treating strictly horizontal defects in inlay/onlay protocols [9].

## 4. Materials and Methods

This systematic review and meta-analysis was performed and reported by following the preferred reporting items for systematic reviews and meta-analysis (PRISMA) guidelines [24].

### 4.1. Search Strategy

A systematic literature review was performed on PubMed, Scopus, EMBASE electronic database through the keywords: synthetic blocks, synthetic bone blocks, block graft, bone defects, bone regeneration, reconstruction procedures, as the search paradigm, with no restrictions regarding publication date. Appropriate search strategies were set for each database. The study pool was then restricted to in vivo studies in which scaffold block grafting was reported. The last search was performed on April 15, 2019. The reference list of the included studies and reviews was also evaluated to find additional eligible articles not retrieved by the electronic search.

### 4.2. Inclusion Criteria

In vivo comparative studies assessing the outcome of synthetic block scaffold in bone regenerative procedures were included. According to the search criteria, the experimental research had to include study models in which the biomaterial was applied for the treatment of any type of bone defects. In vitro studies were excluded, but if they also reported an in vivo study, they were taken into consideration. No limitations about the type of synthetic blocks were applied to the research. Studies that did not comply with the inclusion criteria, reviews, clinical case reports, case series, and in vitro studies were excluded from the evaluation.

### 4.3. Selection of the Studies 

The initial screening of the studies was performed independently by two experienced reviewers (M.D.F. and M.T.) based on title and abstract. In the cases where abstracts were not available, the full text was obtained and checked. Publications not conforming to the selection criteria were excluded. Full text of all eligible articles was obtained and checked for inclusion criteria. For excluded articles, the reasons for exclusion were recorded.

### 4.4. Data Extraction

Data from included articles were extracted and analyzed independently by the two authors, following a specially designed data-collection form, which ensured systematic recording of data. The aim was to evaluate quantitatively and qualitatively the outcome of the synthetic biomaterials blocks in bone regenerative procedures. The primary outcomes were the percentage of new bone formation and the percentage of residual bone. The secondary outcomes were the bone density at radiographic analysis, the soft tissue health, and the postoperative complications. Other data were the sample size, the gender, the duration of follow-up, the bone defect size and location, and the type of biomaterial used in the test and in the control group.

### 4.5. Risk-of-Bias Analysis 

The ARRIVE (Animals in Research: Reporting In Vivo Experiments) guidelines for reporting animal experiments for assessing risk of bias and other methodological criteria were used to evaluate the risk of bias of the animal studies included in this review. As no human study was found, no specific risk-of-bias assessment tool for clinical trials was applied.

Quality criteria taken into consideration were as follows:

1. Ethical statement (nature of ethical review permissions and national or institutional guidelines for the care and use of animals)
2. Experimental procedures (precise details of all procedures performed)
3. Experimental animals (details of animal used, including species, developmental stage or mean age, type of teeth, and diagnosis)
4. Randomization
5. Allocation concealment
6. Blinding of the evaluator
7. Sample size calculation
8. Completeness of information on dropouts
9. Statistical analysis appropriateness
10. Financial conflict of interest

The evaluation of the methodological quality of the selected studies was performed by the reviewers. All the criteria were assessed as adequate, unclear, or inadequate. The studies with at least 7/10 appropriate parameters and no inappropriate parameters were considered as low. Otherwise, the studies were classified as high risk. The risk-of-bias analysis was performed using the software RevMan Version 5.3 (The Nordic Cochrane Centre, The Cochrane Collaboration, Copenhagen, 2014).

### 4.6. Meta-Analysis Inclusion Criteria 

It was planned to perform a meta-analysis of the studies that compared a synthetic block vs. an autologous block or a synthetic block vs. spontaneous healing. All articles that did not respect such criteria were not included in the meta-analysis. The meta-analysis was performed only for studies with similar comparisons reporting the same outcome measures. Mean differences were combined for continuous data, using random-effects models if at least four studies were included in the meta-analysis, while if there were less than four studies, a fixed-effects model was chosen. For split-mouth studies, an intraclass correlation coefficient equal to zero was assumed. Also, when possible, subgroups analysis was performed for studies presenting results at different time frames. The meta-analysis was performed using the software RevMan Version 5.3. The outcome variable considered for the meta-analysis was the percentage of new bone formation in test and control sites with the use of a histomorphometric analysis.

## 5. Conclusions

In conclusion, synthetic bone blocks represent a viable resource in bone regenerative surgery to achieve new bone formation and minimize the risks of infection transmission. The physical and biological properties of the synthetic scaffold seem to improve the new bone formation in experimental defects in vivo. The advances in modern biomaterials science may allow enhancing the predictability of regenerated bone quality and quantity for the treatment of maxillary bony defect.

## Figures and Tables

**Table 1:** General features of the 18 studies performed in a rabbit study model.

Authors	N. Animals	Defect	Test Block	Control Site	Analysis Time	Evaluation
**Studies on Rabbit**						
Torres et al. 2011 [25]	8	Calvaria	Monetite in different thickness	-	8 weeks	Histology and Histomorphometry
Zivadinovic et al. 2016 [26]	8	Calvaria	β-tricalcium phosphate	Unfilled, Autologous graft	4 weeks	Histology and Histomorphometry
Tamimi et al. 2009 [27]	8	Calvaria	Monetite	Autologous graft	8 weeks	Histology and Histomorphometry
Kim et al. 2012 [63]	16	Calvaria	Block-type biphasic calcium phosphate (BCP), rhBMP-2, collagene	-	8 weeks	Histology and Histomorphometry
Hwang et al. 2012 [28]	10	Calvaria	Hydroxyapatite, β-tricalcium phosphate, Biphasic calcium phosphate synthetic block-type bone graft	Unfilled	4–8 weeks	micro-CT, Histology and Histomorphometry
Bae et al. 2014 [29]	16	Calvaria	Hydroxyapatite bone block	Autologous graft	4–8 weeks	micro-CT, Histology and Histomorphometry
Shim et al. 2012 [30]	18	Calvaria	polycaprolactone(PCL)/poly(lactic-co-glycolic acid) (PLGA) scaffold blended with tri-calcium phosphate (TCP)	Unfilled	4–8 weeks	micro-CT, Histology and Histomorphometry
Xu et al. 2008 [31]	12	Calvaria	β-calcium silicate (b-CaSiO3, b-CS), porous β-tricalcium phosphate (b-Ca3(PO4)2; β -TCP	-	4–8 and 16 weeks	micro-CT, Histology and Histomorphometry
Yoo et al. 2017 [32]	7	Calvaria	Biphasic calcium phosphate(BCP); Biphasic calcium phosphate phosphate/carboxymethyl cellulose (BCP/CMC); Biphasic calcium phosphate/cross-linked carboxymethyl cellulose (BCP/c-CMC); Biphasic calcium phosphate/hyaluronic acid (BCP/HyA)	-	4 weeks	micro-CT, Histology and Histomorphometry
Lam et al. 2009 [34]	6	Calvaria	PCL scaffolds (NaOH treated for 12h); Predegraded PCL scaffolds (PD-PCL, NaOH treated for 7 days); Untreated PCL/TCP scaffolds	-	18–24 weeks	Histology
Gehrke et al. 2019 [35]	20	Calvaria	Sintered bovine bone blocks Nonsintered bovine bone blocks	-	6 and 8 weeks	Histology and Histomorphometry
Kanazawa et al. 2017 [61]	19	Femur and tibia defects	Carbonate apatite (CO3Ap); Hydroxyapatite (HA) block	-	4, 12, and 24 weeks	micro -CT
Tsai et al. 2008 [36]	16	Femur defects	CPC (amorphous calcium phosphate, DCPD powders mixed with physiological saline) in different concentration	-	1, 4, 12, and 24 weeks	Histology
Teramoto et al. 2005 [37]	38	Femur defects	β-tricalciumphosphate (75% porosity); Apatite-wollastonite glass-ceramic (70, 80, and 90% porosity)	-	8, 12, 24, and 36 weeks	Histology
Ohsawa et al. 2004 [38]	3 Femur defects	Porous apatite Wollastonite-containing glass-ceramic (AW)	-	3, 6, 12 months	Radiographs, Histology
Guo et al. 2012 [39]	6	Mandible defects (angle and body)	Composite nano-HA/polyamide (n-HA/PA); Composite n-HA/PA+ BMSC bone marrow stromal cells	-	4–12 weeks	Histology, Histomorphometry, SEM, micro-CT
Zhang et al. 2009 [40]	12	Radius defects	Nanocomposite of hydroxyapatite surface-grafted with poly(L-lactide and Poly- Glycolidee (g-HAP)	-	4, 8, 12, and 20 weeks	Radiographs, Histology

**Table 2:** General features of the four studies performed on dogs.

Authors	N. Animals	Defect	Test Block	Control Site	Analysis Time	Evaluation
**Studies on Dogs**						
Rismanchian et al. 2015 [41]	4	Maxillary defects	Bioglass (BG), Demineralized bone matrix (DBM), Forstrite (FR)	Unfilled	15, 30, 45, and 60 days	Histology and Histomorphometrry
Du et al. 2015 [42]	4	Mandibular critical-size defect	Nano Hydroxyapatite (nHA) coral blocks; recombinant human vascular endothelial growth factor165 (rhVEGF), Nano Hydroxyapatite (nHA)/coral blocks	-	3 and 8 weeks	Histology, Histomorphometry
Nery et al. 1992 [43]	21	Mandibular and maxillary periodontal defects (Canines and 1st molar)	Hydroxyapatite/beta tricalcium phosphate (HA/ßTCP) in different macroporosity, Biphasic calcium phosphate ceramic	Unfilled	6 months	Histology, Histomorphometry
Erbe et al. 2001 [44]	4	Cylindrical metaphyseal defects	B-TCP synthetic cancellous bone	-	12, 24, and 52 week	Radiograph,X-ray diffraction (XRD), Histology and Histomorphometry t 12, 24, and 52 week

**Table 3:** General features of the seven studies performed on rats.

Authors	N. Animals	Defect	Test Block	Control Site	Analysis Time	Evaluation
**Studies on Rats**						
Gabbai-Armelin et al. 2015 [45]	60	Tibia defect	Bioactive fibrous glassy scaffold	Unfilled	15, 30, and 60 days	Histology, Histomorphometry
Coraca ¸ et al. 2008 [46]	44	Tibia defect	Poly(l-lactic acid)PLLA/poly(ethylene oxide) PEO blend, poly(l-lactic acid) PLLA	-	2, 4, 6, 8 weeks	Histology, Histomorphometry
Inzana et al. 2014 [47]	18	Femural defect	Calcium phosphate scaffold; CPh scaffold, collagen binder; CPh scaffold collagen coating; Devitalized allograft	Unfilled	9 weeks	Histology, Histomorphometry, SEM evaluation
Hwang et al. 2017 [62]	32	Calvaria	Polycaprolactone(PCL) polylactic-co-glycolic acid PLGA) β-tricalcium phosphate in a 4:4:2 ratio, Biphasic calcium phosphate	-	2, 4, 6, 8 weeks	Histology, Histomorphometry
Montjovent et al. 2007 [48]	24	Calvaria	Bioresorbable scaffolds made of polylactic acid/beta tricalcium phosphate; PLa/Hydroxyapatite; Beta tricalcium phosphate	-	18 weeks	Radiographs, Histology
Hulsart-Billström et al. 2011 [49]	18	Ectopic bone formation	Beta tricalcium phosphate (ß-TCP)/nano hydroxyapatite, hydroxyapatite, Clods of hydroxyapatite, HAP biomimetic	Unfilled	4 weeks	Radiographs, Histology
do Carmo et al. 2017 [50]	20	Maxillary dental socket	Nanostructured carbonated hydroxyapatite/sodium alginate 5% strontium microspheres, Nanostructured carbonated hydroxyapatite/sodium alginate	-	1 and 6 weeks	Histology

**Table 4:** General features of the four studies performed on minipigs.

Authors	N. Animals	Defect	Test Block	Control Site	Analysis Time	Evaluation
**Studies on Minipigs**						
Yeo et al. 2011 [51]	10	Mandible lateral defect	PCL-TCP scaffold, Collagen membrane	Autologous graft+ collagen membrane	24 weeks	micro-CT, Histology and Histomorphometry
Kirchhoff et al. 2011 [52]	6	Mandible defect	Nanostructured hydroxyapatite (HA) porous matrix of silica (SiO2) gel	Autologous graft	5 and 10 weeks	Histology, Histomorphometry
Henkel et al. 2006 [53]	15	Critical-size mandible defects	CaP matrix (HA:TCP = 60%:40%); Monophasic CaP matrix (HA: 100%), Pure hydroxyapatite; Beta tricalcium phosphate Gelatin sponge	-	32 weeks	Histology, Histomorphometry
Verket et al. 2016 [54]	5	Implants with dehiscence defects	TiO_2_ scaffold	Autologous graft	12 weeks	Histology, Histomorphometry

**Table 5:** General features of the four studies performed on goats.

Authors	N. Animals	Defect	Test Block	Control Site	Analysis Time	Evaluation
**Studies on Goats**						
Nandi et al. 2009 [55]	12	Radius defects	Porous bioactive glass blocks	Unfilled	90 days	Histology, Radiographs and Angiogram
Habibovic et al. 2008 [56]	12	Lumbar transverse processes	BCPA, BCPB, BCPC, one composite (BCPCþ) of BCPC reinforced with PLA, HA, and CA ceramic.	-	3, 6, 9, and 12 weeks	Histology, Histomorphometry
Habibovic et al. 2008 [57]	12	Transverse processes (L1–L4)	Ceramic tricalcium phosphate (TCP) brushite, monetite	-	3, 6, and 9 weeks	Histology, and Histomorphometry, SEM evaluation
Koeter et al. 2008 [58]	20	Knee defects	Coralline hydroxyapatite (CHA)	Autologous bone	12 weeks	Histology, Histomorphometry

**Table 6:** General features of the two studies performed on sheep.

Authors	N. Animals	Defect	Test Block	Control Site	Analysis Time	Evaluation
**Studies on Sheep**						
von Doernberg et al. 2006 [59]	9	Methaphysial or epiphysial long bones	Beta-TCP cylinders at different pore size	Unfilled	90 days	Histology, Radiographs and Angiogram
Gosain et al. 2004 [60]	10	Calvaria	60% hydroxyapatite and 40%-TCP; 60% hydroxyapatite–cement and 40%-TCP; 20% hydroxyapatite–cement and 80%-TCP; Pure hydroxyapatite;	-	3, 6, 9, 12 weeks	Histology, Histomorphometry

**Table 7:** Synthesis of the histomorphometric results relative to new bone formation (NBF) at four and eight weeks of healing in the included studies that provided such information. Only studies comparing a synthetic block versus unfilled control defect were considered. Only the five studies on rabbits were submitted to meta-analysis.

Author	N. TEST	Model TEST	Mean% NBF TEST	SD TEST	N CTR	Mean% NBF CTR	SD CTR	CTR	Mean Difference% (95% CI)
Zivadinovic et al. 2016 [26]	8	Rabbit (β-TCP)	30.15% (4 weeks)	5.71	8	7.32 % (4 weeks)	8.40	UNFILLED	22.83 (15.79, 29.87)
Tamimi et al. 2009 [27]	8	Rabbit (monetite)	43.4% (8 weeks)	8.1	8	60.1% (8 weeks)	6.0	AUTOLOGOUS	−16.30 (−23.29, −9.31)
Hwang et al. 2012 [28]	5	Rabbit (HA)	4.97% (4 weeks)	1.91	5	3.99% (4 weeks)	1.54	UNFILLED	0.98 (−1.17, 3.13)
	5	Rabbit (β-TCP)	1.56% (4 weeks)	1.32					−2.43 (−4.21, −0.65)
	5	Rabbit (BCP)	5.60% (4 weeks)	3.93					1.61 (−2.09, 5.31)
	5	Rabbit (HA)	6.95% (8 weeks)	3.51	5	4.55% (8 weeks)	2.50	UNFILLED	2.40 (−1.38, 6.18)
	5	Rabbit (β-TCP)	4.04% (8 weeks)	1.39					−0.51 (−3.02, 2.00)
	5	Rabbit (BCP)	9.03% (8 weeks)	3.39					4.48 (0.79, 8.17)
Shim et al. 2012 [30]	6	Rabbit (PCL/PLGA)	10.74% (4 weeks)	1.86	6	4.06% (4 weeks)	2.03	UNFILLED	6.68 (4.48, 8.88)
	6	Rabbit (PCL/PLGA/TCP)	14.29% (4 weeks)	4.59					10.23 (6.21, 14.25)
	6	Rabbit (PCL/PLGA)	15.68% (8 weeks)	2.89	6	10.08% (8weeks)	1.86	UNFILLED	5.60 (2.85, 8.35)
	6	Rabbit (PCL/PLGA/TCP)	20.75 % (8 weeks)	4.20					10.67 (6.99, 14.35)
Rismanchian et al. 2015 [41]	4	Dog (BG)	22.65% (8 weeks)	2.76	4	23.43% (8 weeks)	5.26	UNFILLED	−0.78 (−6.60, 5.04)
	4	Dog (FR)	26.65% (8 weeks)	4.51					3.22 (-3.57, 10.01)
	4	Dog (BG)	21.21 (4 weeks)	0.94	4	22.37 (4 weeks)	3.44	UNFILLED	−1.16 (−4.65, 2.33)
	4	Dog (FR)	26.56 (4 weeks)	6.97					4.19 −3.43, 11.81)
Gabbai-Armelin et al. 2015 [45]	60	Rat (BG)	21.3 % (8 weeks)	2.4	60	46.8% (8 weeks)	7.1	UNFILLED	−25.50 (−27.40, −23.60)
Gosain et al. 2004 [60]	10	Sheep (60%HA-CC)	13.6% (1 year)	2.0	10	0 (1 year)	0	UNFILLED	N.E.
	10	Sheep (60%HA-CP)	11.2% (1 year)	2.3					N.E.
	10	Sheep (20%HA-CP)	28.5% (1 year)	4.5					N.E.
	10	Sheep (100%HA-CP)	4.8% (1 year)	1.4					N.E.
Kirchhoff 2010 [52]	6	Minipig (nHA-A)	7.6% (5 weeks)	6.0	-	-	-	NO CONTROL	N.E.
	6	Minipig (nHA-B)	15.3% (5 weeks)	8.3					N.E.
	6	Minipig (nHA-A)	34.1% (10 weeks)	10.8					N.E.
	6	Minipig (nHA-B)	39.9% (10 weeks)	13.5					N.E.
